# A novel lnc-PCF promotes the proliferation of TGF-*β*1-activated epithelial cells by targeting miR-344a-5p to regulate map3k11 in pulmonary fibrosis

**DOI:** 10.1038/cddis.2017.500

**Published:** 2017-10-26

**Authors:** Huizhu Liu, Bingsi Wang, Jinjin Zhang, Songzi Zhang, Youlei Wang, Jie Zhang, Changjun Lv, Xiaodong Song

**Affiliations:** 1 School of Pharmaceutical Sciences, Binzhou Medical University, Yantai 264003, China; 2 School of Pharmaceutical Sciences, Taishan Medical University, Taian 271016, China; 3 Department of Respiratory Medicine, Affiliated Hospital to Binzhou Medical University, Binzhou 256602, China

## Abstract

Emerging evidence suggests that microRNA (miRNA) and long noncoding RNA (lncRNA) play important roles in disease development. However, the mechanism underlying mRNA interaction with miRNA and lncRNA in idiopathic pulmonary fibrosis (IPF) remains unknown. This study presents a novel lnc-PCF that promotes the proliferation of TGF-*β*1-activated epithelial cells through the regulation of map3k11 by directly targeting miR-344a-5p during pulmonary fibrogenesis. Bioinformatics and *in vitro* translation assay were performed to confirm whether or not lnc-PCF is an actual lncRNA. RNA fluorescent *in situ* hybridization (FISH) and nucleocytoplasmic separation showed that lnc-PCF is mainly expressed in the cytoplasm. Knockdown and knockin of lnc-PCF indicated that lnc-PCF could promote fibrogenesis by regulating the proliferation of epithelial cells activated by TGF-*β*1 according to the results of xCELLigence real-time cell analysis system, flow cytometry, and western blot analysis. Computational analysis and a dual-luciferase reporter system were used to identify the target gene of miR-344a-5p, whereas RNA pull down, anti-AGO2 RNA immunoprecipitation, and rescue experiments were conducted to confirm the identity of this direct target. Further experiments verified that lnc-PCF promotes the proliferation of activated epithelial cells that were dependent on miR-344a-5p, which exerted its regulatory functions through its target gene map3k11. Finally, adenovirus packaging sh-lnc-PCF was sprayed into rat lung tissues to evaluate the therapeutic effect of lnc-PCF. These findings revealed that lnc-PCF can accelerate pulmonary fibrogenesis by directly targeting miR-344a-5p to regulate map3k11, which may be a potential therapeutic target in IPF.

The majority of genomes are transcribed using modern molecular biology techniques, such as deep sequencing; however, only 2% of the transcribed genome codes are attributed to proteins. The remaining part of the transcribed genome is known as noncoding RNA (ncRNA). ncRNAs can be divided into small (<200 nt) ncRNAs, such as microRNAs (miRNAs) and transfer RNAs, and long RNAs(>200 nt), such as long noncoding RNAs (lncRNAs) and ribosomal RNAs.^1^ Although miRNAs and lncRNAs play important roles in the development and progression of diseases,^2, 3^ the interaction of mRNAs with miRNAs and lncRNAs to form an interrelated regulatory network in diseases remains unknown.

Some lncRNAs regulate the biological function of miRNAs by recruiting the miRNAs as competitive endogenous RNAs (ceRNAs).^4^ These lncRNAs generally exhibit the same miRNA response element with the transcripts of mRNAs. For instance, lnc-MD1 ‘sponges’ miR-133 and miR-135 to regulate the expression of MAML1 and MEF2C, thereby activating muscle-specific gene expression.^5^ lnc-APF is identified as the ceRNA that can regulate autophagic cell death by targeting miR-188-3p and ATG7.^6^ Nevertheless, the mechanism underlying mRNA interaction with miRNA and lncRNA in idiopathic pulmonary fibrosis (IPF) remains unknown. IPF is defined as a specific form of chronic and progressive interstitial pneumonia of an unknown cause, and this disease leads to the progressive loss of lung function, respiratory failure, and death.^7^ To date, lung transplant is the only effective treatment available for IPF.^8^ Thus, a comprehensive understanding of the molecular mechanisms of fibrogenesis is required in developing specific therapies toward this disease.^9^

This paper is a follow-up to a previously completed research in our laboratory. In the previous study, we described the differentially expressed miRNAs, lncRNAs, and mRNAs in IPF; however, the molecular mechanisms of these RNAs are poorly elucidated.^10, 11^ In the present work, we defined a novel lnc-PCF, its function, and the crosstalk between lnc-PCF and the map3k11 target miR-344a-5p in the regulation of fibrogenesis.

## Results

### BC158825 was verified as a novel lncRNA and highly expressed in pulmonary fibrosis

**Figure 1:**
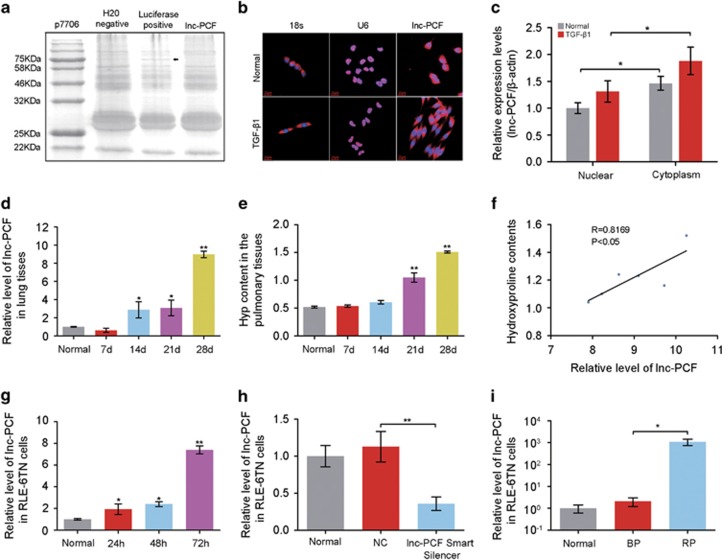
Verification of lnc-PCF as a novel lncRNA and its high expression level in the development of pulmonary fibrosis *in vivo* and *in vitro*. (**a**) *In vitro* translation assay showed that lnc-PCF performed no translation activities. The black arrow indicates that the positive control could translate a 75 kDa protein. (**b**) RNA FISH detecting the location of endogenous lnc-PCF (red) in cells. The result showed that lnc-PCF mainly localized in the cytoplasm. U6 and 18S RNA was used as nuclear and cytoplasmic localization markers, respectively. DNA (blue) was stained with DAPI. (**c**) Nucleocytoplasmic separation result confirmed that lnc-PCF was mainly expressed in the cytoplasm by using qRT-PCR. (**d**) Increased lnc-PCF expression in BLM-treated lung tissues at 0–28 days by using qRT-PCR. (**e**) Hydroxyproline (Hyp) content increased in the rat pulmonary tissues at 0–28 days, and it was higher at 21–28 day BLM-treated groups than that in the normal group. (**f**) Positive correlation was measured between the lnc-PCF expression and Hyp content *in vivo* by using Pearson correlation coefficient. (**g**) Increased lnc-PCF expression in TGF-*β*1-induced rat lung epithelial-t-antigen negative (RLE-6TN) cells from 0 h to 72 h. (**h**) Cells were transfected with lnc-PCF smart silencer /NC. And The smart silencer could significantly inhibit the expression of lnc-PCF. NC indicates a negative control of lnc-PCF smart silencer. (**i**) Recombinant plasmid of overexpressed lnc-PCF could increase the expression level of lnc-PCF. BP indicates blank plasmid. RP indicates recombinant plasmid of the overexpressed lnc-PCF. Each bar represents the mean±S.D., *n*=6. *P<0.05, **P<0.01

Our laboratory studies revealed the different expression levels of lncRNA in pulmonary fibrosis and normal lung tissues by using microarray analysis,^10, 11^ and we also acquired transcript BC158825, which is significantly different and has higher expression than other transcripts. Therefore, we selected BC158825 for further study and renamed it as lnc-PCF (lncRNA promotes proliferation of epithelial cell activated with TGF-*β*1 during pulmonary fibrogenesis) which revealed its function. According to our database, lnc-PCF is a sense lncRNA located at the second chromosome (Supplementary Figure 1a). In actual cases, many predicted lncRNAs are not real lncRNAs because these lncRNAs can still encode proteins.^12^ To verify whether or not lnc-PCF is a real lncRNA, we further analyzed its full-length sequence obtained from the National Center for Biotechnology Information. Data showed that lnc-PCF is a *Rattusnorvegicus* linear cDNA (IMAGE: 7376599) with a length of 1208 bp (Supplementary Figure 1b). To prove that lnc-PCF cannot encode proteins, we divided the full-length sequence into six parts using the open-reading frame (ORF) finder database. Among the six parts, nine sequences indicated with a blue line were considered to possess protein-coding ability. However, none of these nine sequences contained >200 nucleotides, and the amino acid that corresponded to these sequences could not match any protein in the Pfam database (Supplementary Figure 1c). In addition, the protein-coding potential of lnc-PCF was evaluated by using the coding potential calculator tool (http://cpc.cbi.pku.edu.cn/) and the coding potential assignment tool (http://lilab.research.bcm.edu/cpat/index.php). The protein-coding ability scores of lnc-PCF were -0.720785 and 0.157507, which indicated that lnc-PCF was devoid of protein-coding potential.^13, 14^ Lnc-PCF translation activities were measured in an *in vitro* translation system, which revealed that this gene has no translation activities (Figure 1a). These analyses strongly confirmed the prediction in lnc-PCF microarray and verified that lnc-PCF is an actual lncRNA. Furthermore, the subcellular localization of lnc-PCF was detected by fluorescent *in situ* hybridization (FISH) and confirmed by quantifying nuclear/cytoplasmic RNA. The results showed that the lnc-PCF transcripts were more localized in the cytoplasm than in the nucleus (Figures 1b and c).

To further verify the microarray analysis result, we investigated the expression levels of lnc-PCF for 28 days at a 7-day interval in bleomycin (BLM)-induced animal model by using quantitative real-time PCR (qRT-PCR). The increasing expression level of lnc-PCF was observed at 14, 21, and 28 days, but the level decreased at 7days (Figure 1d), which is considered an inflammatory phase and not fibrosis.^15^ Hydroxyproline (Hyp) is an indicator of pulmonary fibrosis. A significant difference was observed for Hyp level at the given time points compared with that in the sham group (Figure 1e). The highest Hyp expression was observed at 21 and 28 days. Pearson’s correlation coefficient was used to confirm the correlation of lnc-PCF and Hyp. Statistical analysis indicated that lnc-PCF is positively correlated with Hyp, thereby suggesting that lnc-PCF is correlated to the degree of pulmonary fibrosis (Figure 1f).

As the most important regulatory factor among fibrogenic cytokines,^16, 17^ TGF-*β*1 was used to stimulate the rat lung epithelial-t-antigen negative (RLE-6TN) cells to confirm the results *in vitro*. Lnc-PCF was upregulated in TGF-*β*1-induced RLE-6TN cell model as compared with that in the normal group (Figure 1g). These experimental results confirmed the microarray analysis results, which stated that lnc-PCF is a confirmed lncRNA that is highly expressed in pulmonary fibrosis. These data suggested that lnc-PCF may play an important role in pulmonary fibrosis.

The interfering sequence of lnc-PCF (lnc-PCF smart silencer, knockdown lnc-PCF), which targeted six different sections of lnc-PCF, and the recombinant plasmid (pLenti-EF1a-EGFP-F2A-Puro-CMV-MCS) of the overexpressed lnc-PCF (RP-lnc-PCF, knockin lnc-PCF) were designed and transfected to RLE-6TN cells to investigate and confirm the function of lnc-PCF in pulmonary fibrosis. The efficiency of knockdown and knockin lnc-PCF was subsequently evaluated by qRT-PCR. The lnc-PCF smart silencer effectively interfered with lnc-PCF expression (Figure 1h). Meanwhile, RP-lnc-PCF induced high levels of lnc-PCF in RLE-6TN cells (Figure 1i).

### Lnc-PCF promoted fibrogenesis in pulmonary fibrosis

**Figure 2:**
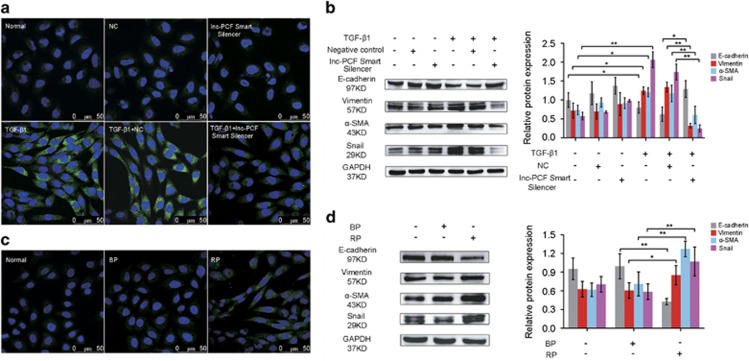
Lnc-PCF promoted fibrogenesis *in vitro*. (**a**) *α*-SMA, which is a physiological marker of fibrogenesis, was observed under a laser scanning confocal microscope. The lnc-PCF smart silencer can reduce *α*-SMA expression compared with TGF-*β*1-treated+NC groups. *α*-SMA around the nuclear membrane was stained with FITC (green). Nuclei were counterstained with Hoechst 33258 (blue). (**b**) Lnc-PCF smart silencer could reduce the expression levels of *α*-SMA, Snail, and vimentin and increase the E-cadherin expression level, as shown by the western blot analysis. RLE-6TN cells were first transfected with 50 nM lnc-PCF smart silencer/NC for 6 h and subsequently cotreated with 5 ng/ml TGF-*β*1 for 72 h. (**c**) Knockin lnc-PCF (RP) can increase the *α*-SMA, as shown by the laser scanning confocal microscope observation. (**d**) Knockin lnc-PCF can increase the expression levels of *α*-SMA, Snail, and vimentin and decrease the E-cadherin expression levels, as shown in the western blot analysis. RLE-6TN cells were first transfected with 2 *μ*g of 1 *μ*g/ml lnc-PCF BP/RP for 6 h and then cotreated with 5 ng/ml TGF-*β*1 for 72 h. NC indicates a negative control of lnc-PCF smart silencer, BP indicates blank plasmid, and RP indicates the recombinant plasmid of the overexpressed lnc-PCF. Each bar represents the mean±S.D., *n*=6, *P<0.05, **P<0.01

During pulmonary fibrosis, the epithelial cells obtain mesenchymal characteristics, including the expression of mesenchymal markers, such as *α*-smooth muscle actin (*α*-SMA), vimentin, transcription repressor Snail, and loss of the epithelial cell marker E-cadherin.^18, 19^ The protein levels of these markers were examined after incubation with TGF-*β*1 for 72 h under knockdown or knockin lnc-PCF by using immunofluorescence staining and western blot analysis to investigate the effect of lnc-PCF on fibrogenesis. Immunofluorescence data showed that the *α*-SMA level was significantly higher in the cells treated with TGF-*β*1 alone than in the other groups. Nonetheless, *α*-SMA expression reached lower levels in the cells treated with lnc-PCF smart silencer +TGF-*β*1 than in TGF-*β*1+NC group (Figure 2a). Western blot analysis further confirmed that the effects of lnc-PCF smart silencer on *α*-SMA expression (Figure 2b). Lnc-PCF smart silencer can reduce the expression levels of vimentin and Snail and increase that of E-cadherin (Figure 2b). By contrast, lnc-PCF overexpression can promote the expression levels of vimentin, Snail, and *α*-SMA and inhibit E-cadherin expression (Figures 2c and d). These findings indicated that knockin lnc-PCF could promote fibrogenesis, whereas knockdown lnc-PCF could block fibrogenesis in pulmonary fibrosis.

### Lnc-PCF promoted the proliferation of epithelial cell activated with TGF-*β*1

**Figure 3:**
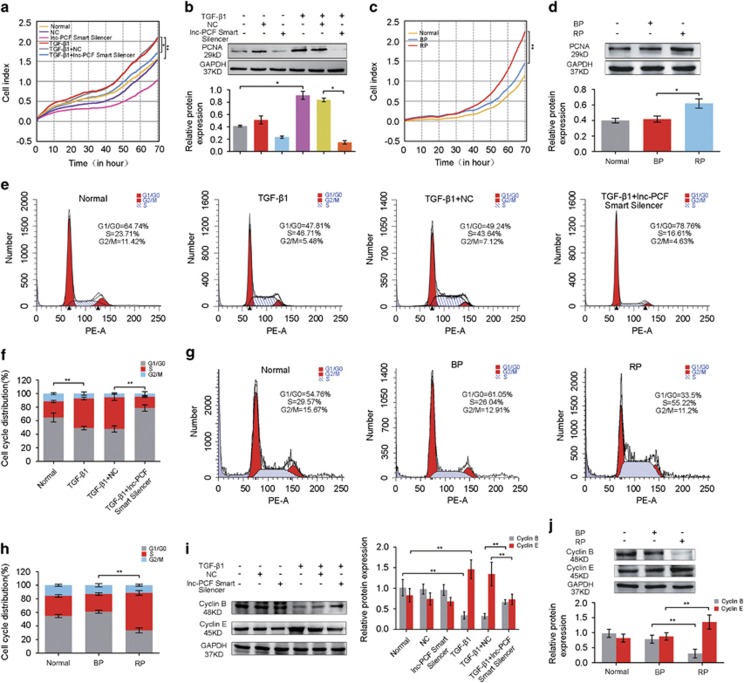
lnc-PCF promoted the proliferation of epithelial cells activated with TGF-*β*1. (**a**) lnc-PCF smart silencer evidently inhibited cell proliferation compared with the TGF-*β*1+NC group. Proliferation analysis was performed using an xCELLigence real-time cell analysis (RTCA) system. (**b**) PCNA protein levels were analyzed by Western blot analysis. The lnc-PCF smart silencer evidently retarded PCNA expression compared with that in the TGF-*β*1+NC group. (**c**) Knockin lnc-PCF could promote cell proliferation compared with the normal control group. Proliferation analysis was performed using a RTCA system. (**d**) Knockin lnc-PCF could promote the proliferating cell nuclear antigen (PCNA) expression compared with that of the normal control group by western blot analysis. (**e**) Cell cycle was analyzed by flow cytometry. Cells were pretreated with 50 nM lnc-PCF smart silencer/NC for 6 h and then exposed to 5 ng/ml TGF-*β*1 for 72 h. The DNA in RLE-6TN cells were labeled with propidium iodide (PI). (**f**) Percentage histogram of cell cycle distribution was presented according to flow cytometry analysis. Lnc-PCF smart silencer increased the percentage of cells in the G1/G0 phase and decreased that in S and G2/M. (**g**) Cell cycle was analyzed by flow cytometry. Cells were pretreated with 2 *μ*g of BP/RP for 6 h and subsequently exposed to 5 ng/ml TGF-*β*1 for 72 h. The DNA in RLE-6TN cells was labeled with PI. (**h**) Percentage histogram of cell cycle distribution was presented according to flow cytometry analysis. Knockin lnc-PCF reduced the percentage of cells in the G1/G0 phase and increased that in S and G2/M. (**i**) Lnc-PCF smart silencer decreased and increased the expression levels of cyclin E and cyclin B, respectively, as shown by western blot analysis. (**j**) Knockin lnc-PCF increased and inhibited the expression levels of cyclin E andcyclin B, respectively, as shown by western blot analysis. NC indicates a negative control of lnc-PCF smart silencer, BP indicates blank plasmid, and RP indicates the recombinant plasmid of overexpressed lnc-PCF. Each bar represents the mean±S.D., *n*=6, *P<0.01, **P<0.05

Pulmonary fibrosis is a neoproliferative disorder of the lung and exhibits uncontrolled proliferation that is similar to the cell growth in cancer.^20, 21^ Thus, we investigated the involvement of lnc-PCF in controlling cell proliferation during fibrogenesis. Growth curves showed that the lnc-PCF smart silencer inhibited the proliferation of the cells activated by TGF-*β*1 (Figure 3a). Western blot analysis performed on RLE-6TN cells showed that the levels of proliferating cell nuclear antigen (PCNA) protein were lower in the lnc-PCF smart silencer+TGF-*β*1 group than in TGF-*β*1+NC group (Figure 3b). Moreover, lnc-PCF overexpression significantly promoted cell proliferation and PCNA expression (Figures 3c and d). To further understand the role of lnc-PCF, we selected the cell cycle that was closely associated with cell proliferation to study the function of lnc-PCF during fibrogenesis.^22, 23^ Flow cytometric analysis revealed that lnc-PCF smart silencer significantly increased the percentage of cells in the G1/G0 phase and decreased that in the S and G2/M phases as compared with those in the TGF-*β*1-treated cells (Figures 3e and f). However, overexpressed lnc-PCF reduced the percentage of cells in the G1/G0 phase and increased that in the S and G2/M phases as compared with those in BP group (Figures 3g and h). These data indicated that interfering lnc-PCF expression could induce cell cycle arrest at the G1/G0 phase.

To confirm the above finding, we further evaluated the cell cycle relative proteins of cyclin E and cyclin B by western blot analysis. The lnc-PCF knockdown downregulated the expression of cyclin E, which is essential to allow the cells to pass through the G1/S checkpoint, and upregulated the expression of cyclin B, which was degraded after the G2/M phase (Figure 3i). The lnc-PCF knockdown can inhibit cyclin E activity and cyclin B degradation, which might inhibit the cells from passing through the checkpoint at the G1/S phase. In addition, the effect of lnc-PCF overexpression on RLE-6TN cell cycle was assessed. Lnc-PCF overexpression could promote the cell cycle process in RLE-6TN cells (Figure 3j). All these results suggested that lnc-PCF can promote the proliferation of epithelial cells activated by TGF-*β*1 in pulmonary fibrosis.

### Lnc-PCF directly targeted miR-344a-5p

**Figure 4:**
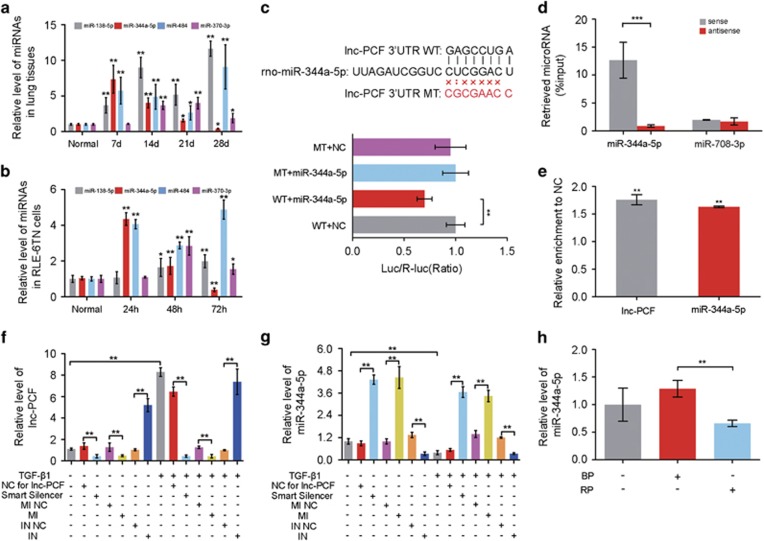
Lnc-PCF directly targeted miR-344a-5p. (**a**) Relative expression of predicted target miRNAs, including miR-344a-5p, miR-370-3p, miR-484, and miR-138-5p, at 0–28 days in the lung tissues of rat pulmonary fibrosis. The miR-344a-5p expression trend was opposite to the lnc-PCF expression *in vivo*. (**b**) Relative expression of predicted target miRNAs, including miR-344a-5p, miR-370-3p, miR-484, and miR-138-5p, at 0–72 h in RLE-6TN cells. The miR-344a-5p expression trend was opposite to the lnc-PCF expression in RLE-6TN cells. (**c**) Cells were transfected with a plasmid vector containing lnc-PCF luciferase reporter, including wild-type (WT) 3′-UTR (5′-GAGCCUGA-3′), mutant-type (MT) 3′-UTR (5′-CGCGAACC-3′), and miR-344a-5p mimic. Cells were transfected for 48 h and collected for luciferase activity assay. Lnc-PCF luciferase activity decreased with cotransfection with miR-344a-5p in WT. (**d**) RNA pull down experiment showed that lnc-PCF was significantly enriched for miR-344a-5p compared with that in non-targeting miR-708-3p. (**e**) RNA immunoprecipitation results showed that endogenous lncRNA-ATB pull down by AGO2 was specifically enriched in miR-344a-5p-transfected cells. (**f**) miR-344a-5p mimic (MI) significantly decreased the lnc-PCF expression. miR-344a-5p inhibitor (IN) promoted the lnc-PCF expression. (**g**) Lnc-PCF smart silencer significantly increased the miR-344a-5p. MI indicates miR-344a-5p mimic, and IN indicates miR-344a-5p inhibitor. RLE-6TN cells were first transfected with 50 nM lnc-PCF smart silencer/NC for 6 h and subsequently cocultured with 5 ng/ml TGF-*β*1 for 72 h. Cells were transfected with 50 nM miR-344a-5p mimic/inhibitor/NC for 6 h and then exposed to TGF-*β*1 treatment for 72 h. (**h**) Knockin lnc-PCF could inhibit the miR-344a-5p expression level. BP indicates blank plasmid, and RP indicates recombinant plasmid of overexpression lnc-PCF. Each bar represents the mean±S.D., *n*=6, *P<0.05, **P<0.01, ***P<0.01

Targeting miRNA is one of the regulatory functions of lncRNA. Therefore, the target miRNA for lnc-PCF was first predicted based on TargetScan, miRanda data and miRbase. Lnc-PCF possesses many predicted target miRNAs that are possibly the regulatory factors of lnc-PCF. Thus, the bioinformatics of lnc-PCF and predicted target miRNAs was used to evaluate their affinity to limit the selection. The selection rules are as follows: (1) in a 2D structure that contains the binding site position of 3′-UTR or full-length sequence of lncRNA, miRNA, and lnc-PCF should be completely paired. The binding type includes 8mer, 7mer-m8, 7mer-al, 6mer, offset 6mer, and imperfect (nonstandard, mismatch or deficiency in G/U pair). Except for imperfect type, other types possess approximately the total number of paired nucleotides. Seed match is not required if the binding type is imperfect. (2) Many red bars that mark the AU weight located on both sides of the seed sequence are preferable. (3) Binding sites that are close to both sides are suitable. MiR-344a-5p, miR-138-5p, miR-370-3p, and miR-484 were selected for the study according to their relative high affinity between lnc-PCF and miRNAs (Supplementary Figure 2). These miRNAs at different time points were evaluated through qRT-PCR acquired from rat pulmonary tissues and RLE-6TN cells to investigate the association of lnc-PCF with the expression trend of these four miRNAs. On one hand, the expression trend of miR-138-5p, miR-370-3p, and miR-484 was not ideal. On the other hand, miR-344a-5p exhibited an opposing expression trend to lnc-PCF *in vivo* and *in vitro* (Figures 4a and b), thereby indicating its potential as a target for lnc-PCF. Thus, miR-344a-5p was selected for further study.

To investigate whether or not miR-344a-5p is targeted by lnc-PCF, we conducted dual-luciferase report system to construct a plasmid vector with full-length lnc-PCF containing wild-type (WT) and mutant-type (MT) 3′-UTR, which was behind two luciferases, namely, firefly and *Renilla*. The luciferase genetic testing report showed that miR-344a-5p overexpression suppressed the luciferase activity of the WT reporter vector but not that of the mutant reporter vector (Figure 4c). This result suggested that miR-344a-5p is a target gene for lnc-PCF.

RNA pull down, anti-AGO2 RNA immunoprecipitation (RIP), and rescue experiments were conducted to further verify the direct targeted relationship between lnc-PCF and miR-344a-5p. RNA pull-down was performed to detect the endogenous miR-344a-5p associated with lnc-PCF using transcribed biotin-labeled lnc-PCF *in vitro*. qPCR analysis revealed that lnc-PCF was significantly enriched in miR-344a-5p as compared with that in non-targeting miR-708-3p (Figure 4d). Moreover, we induced anti-AGO2 RIP and transiently overexpressed miR-344a-5p in RLE-6TN cells. The endogenous lncRNA-ATB pulled down by AGO2 was specifically enriched in the miR-344a-5p-transfected cells (Figure 4e). To investigate the association of lnc-PCF with the expression trend of miR-344a-5p, we evaluated the level of miR-344a-5p at different time points through RT-PCR acquired from rat pulmonary tissues and TGF-*β*1-treated RLE-6TN cells. The miR-344a-5p overexpression (mimic) resulted in decreased lnc-PCF expression, and the suppression of miR-344a-5p (inhibitor) significantly enhanced the lnc-PCF expression as compared with that in the TGF-*β*1+NC group (Figure 4f). In addition, the miR-344a-5p expression levels increased when lnc-PCF was knocked down (Figure 4g) but was reduced by the knockin of lnc-PCF (Figure 4h). All these results revealed the direct targeted association between lnc-PCF and miR-344a-5p.

### Lnc-PCF promoted the proliferation of activated epithelial cells dependent on miR-344a-5p

**Figure 5:**
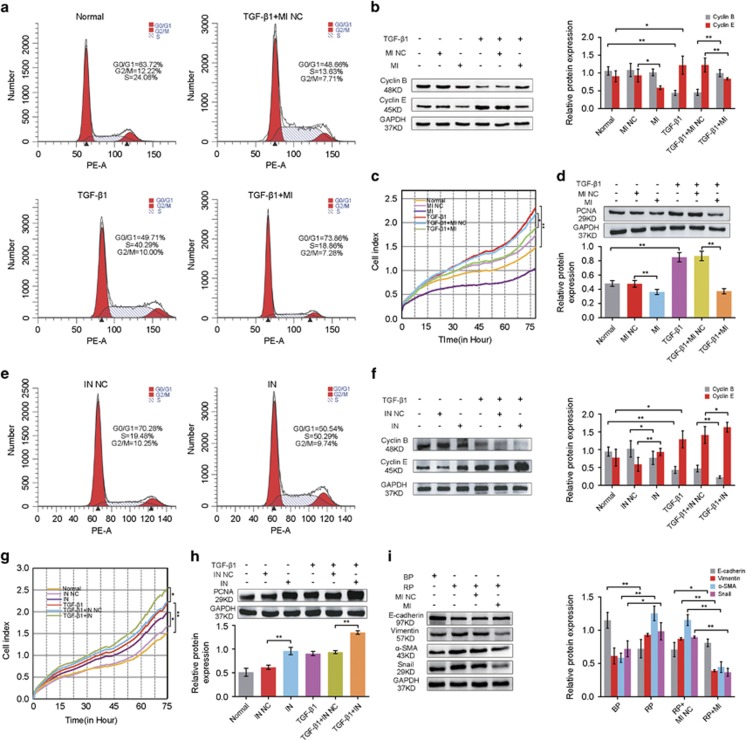
Lnc-PCF promoted cell proliferation dependent on miR-344a-5p. (**a**) The miR-344a-5p mimic (MI) increased the percentage of cells in G0/G1, whereas the percentage of cells decreased in the S and G2/M phases in RLE-6TN cells. RLE-6TN cells were first transfected with 50 nM miR-344a-5p mimic/NC and cocultured with 5 ng/ml TGF-*β*1 for 72 h. The DNA of RLE-6TN cells was labeled with PI and analyzed by flow cytometry. (**b**) miR-344a-5p mimic downregulated cyclin E and upregulated cyclin B expression. (**c**) Proliferation analysis was performed using the RTCA system under the condition of miR-344a-5p mimic transfection. The miR-344a-5p overexpression evidently retarded cell proliferation in RLE-6TN cells. (**d**) PCNA protein levels were tested using western blot analysis. The miR-344a-5p mimic decreased the PCNA expression evidently. (**e**) miR-344a-5p inhibitor (IN) can increase the S and G2/M phase proportion and decrease the G0/G1 phase ratio. RLE-6TN cells were transfected with 50 nM miR-344a-5p inhibitor/NC before the cells were exposed to 5 ng/ml TGF-*β*1 treatment. (**f**) miR-344a-5p inhibitor downregulated cyclin E and increased cyclin B protein expression. (**g**) Proliferation analysis was performed using the RTCA system under the condition of miR-344a-5p inhibitor transfection. Silencing of miR-344a-5p evidently promoted cell proliferation in RLE-6TN cells. (**h**) PCNA protein level was tested by western blot analysis. The miR-344a-5p inhibitor evidently increased the PCNA expression. (**i** and **j**) miR-344a-5p mimic could rescue E-cadherin expression inhibited by lnc-PCF overexpression and decrease *α*-SMA, vimentin, and snail, which were promoted by lnc-PCF overexpression. Each bar represents the mean±S.D., *n*=6,*P<0.05, **P<0.01

Given that lnc-PCF was confirmed to directly target miR-344a-5p, experiments were designed to verify whether or not lnc-PCF promotes the proliferation of activated epithelial cells dependent on miR-344a-5p. The miR-344a-5p mimic and inhibitor were transfected into RLE-6TN cells. Cell proliferation profiles were determined by flow cytometry, western blot analysis, and real-time cell analysis system. Data showed that due to the upregulated miR-344a-5p (mimic) expression, the cells accumulated in G0/G1, and the percentage of cell accumulation in the S and G2/M phases is lower than that in the control (Figure 5a). Western blot analysis showed that the miR-344a-5p overexpression (mimic) could reduce cyclin E expression and increase cyclin B expression (Figure 5b). Cyclin E downregulation can prevent the cells from passing through the G0/G1 checkpoint and increase the expression of cyclin B, which was not degraded during G2/M because of G0/G1 retardation. miR-344a-5p mimic could reduce the growth of activated epithelial cells (Figure 5c) and PCNA (Figure 5d). By contrast, miR-344a-5p inhibitor increased the S and G2/M phase proportion, decreased the G0/G1 phase ratio, downregulated cyclin E, and upregulated cyclin B and PCNA, thereby activating epithelial cell proliferation (Figures 5e and h). We further used a rescue experiment (lnc-PCF overexpression+miR-344a-5p mimic) to detect whether or not the effect of lnc-PCF is dependent on miR-344a-5p. As shown in Figure 5i, the miR-344a-5p mimic rescued the function of lnc-PCF in lung fibrosis and the expression of E-cadherin expression, which was inhibited by lnc-PCF overexpression, and reduced the expression of *α*-SMA, vimentin, and Snail, which were promoted by lnc-PCF overexpression. All of these findings revealed that lnc-PCF promotes cell proliferation that is dependent on miR-344a-5p by directly targeting miR-344a-5p.

### Identification of target-regulating mechanism in miR-344a-5p, map3k11, and lnc-PCF

miRNAs exert their regulatory functions through specific interactions with their target genes. Therefore, the miR-344a-5p target genes were predicted based on TargetScan, miRanda data, and miRbase. The affinity of predicted target genes with miR-344a-5p was also analyzed. Among the list of miR-344a-5p target genes, we focused on map3k11 because it has relatively higher affinity than other genes and is an important indicator of the master regulatory factor in cell differentiation, proliferation, and individual development.^24^

**Figure 6:**
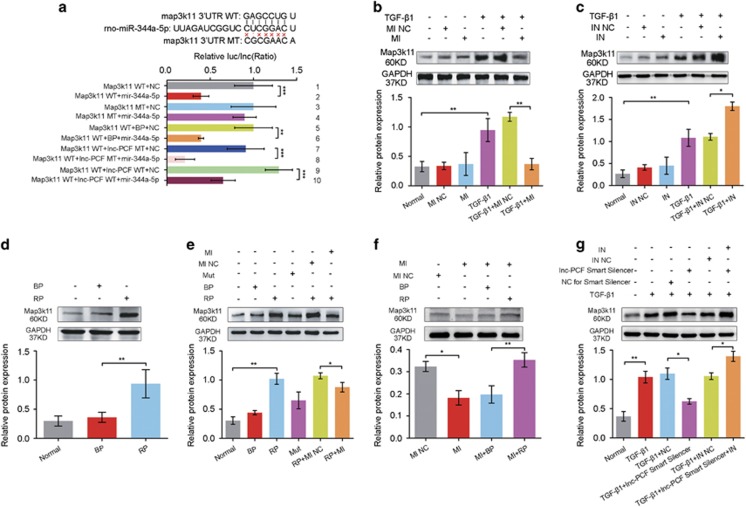
Identification of target-regulating mechanism in miR-344a-5p, map3k11, and lnc-PCF. (**a**) Map3k11 3′-UTR luciferase activities assay. Two types of 3′ UTR (WT: 5′-GAGCCUGU-3′ MT: 5′-CGCGAACA-3′) were cloned for the miR-344a-5p seed sequence (5′-CUCGGACU-3′) and tested for their luciferase activity, as regulated by miR-344a-5p. The luciferase activity of the WT 3′-UTR–map3k11 was significantly decreased in cells transfected with miR-344a-5p mimics, whereas miR-344a-5p mimics could not inhibit the luciferase activities of the MU 3′UTR–map3k11 (1–4). Lnc-PCF could protect map3k11 by competitive binding to miR-344a-5p (5–10). WT indicates wild type, MT indicates mutant type, NC indicates negative control, and BP indicates blank plasmid. (**b**) Western blot analysis showed that map3k11 protein levels decreased after miR-344a-5p mimic (MI) transfection. Protein samples were obtained from RLE-6TN cells after being transfected with miR-344a-5p mimic for 24 h and then exposed to TGF-*β*1 for 72 h. (**c**) Western blot analysis showed that map3k11 protein levels increased after miR-344a-5p inhibitor (IN) transfection. Protein samples were obtained from RLE-6TN cells after being transfected with miR-344a-5p inhibitor for 24 h and then exposed to TGF-*β*1 for 72 h. (**d**) Knockin lnc-PCF could promote map3k11expression. (**e**) miR-344a-5p mimic (MI) could block map3k11 expression increased by overexpression of lnc-PCF. After transfection with RP/BP for 24 h, cells were then cotransfected with miR-344a-5p mimic (MI) for 48 h. Mut indicates mutation binding site with miR-344a-5p in lnc-PCF. (**f**) Knockin lnc-PCF could increase the map3k11 expression, which was blocked with miR-344a-5p mimic (MI). After transfection with miR-344a-5p mimic (MI) for 24 h, cells were then cotransfected with RP/BP for 48 h. (**g**) miR-344a-5p inhibitor (IN) could increase the map3k11 expression by lnc-PCF smart silencer. Data are shown as mean±S.D. *P< 0.05, **P<0.01, ***P<0.01

A luciferase assay reporter system was established by amplifying and inserting the 3′ UTR of map3k11 into a vector containing a downstream firefly luciferase to verify map3k11 as a target gene of miR-344a-5p. The luciferase activity of the WT 3′-UTR–map3k11 was significantly decreased in the cells transfected with miR-344a-5p mimics, whereas the miR-344a-5p mimic could not inhibit the luciferase activities of the MU 3′UTR–map3k11 (Figures 6a, 1–4). Thus, map3k11 is the target gene of miR-344a-5p. In addition, cells were transfected with lnc-PCF WT/MT to confirm that lnc-PCF could protect map3k11 by competitively binding with miR-344a-5p (Figure 6a, 5–10). The miR-344a-5p mimic and inhibitor were transfected into pulmonary epithelial cells activated with TGF-*β*1 to further confirm the target relation between map3k11 and miR-344a-5p. Data showed that the miR-344a-5p mimic could inhibit map3k11 expression, whereas the miR-344a-5p inhibitor could promote map3k11 expression as compared with that in the TGF-*β*1-activated group (Figures 6b and c). The map3k11 levels were inversely correlated with miR-344a-5p expression. Rescue experiments further supported the evidence that miR-344a-5p directly regulates map3k11 expression. Knockdown or knockin of lnc-PCF could significantly downregulate or upregulate the expression of map3K11, which was rescued by cotransfection with the miR-344a-5p inhibitor or mimic (Figures 6d–g). These results indicated that miR-344a-5p directly binds with map3k11, which consequently downregulates its expression level. This occurrence may imply that lnc-PCF competitively binds with miR-344a-5p to protect map3k11 from being degraded by miR-344a-5p, which results in the promotion of cell proliferation. In conclusion, the profibrotic function of lnc-PCF could be mediated by targeting map3K11 via miR-344a-5p.

### Therapeutic value of lnc-PCF *in vivo*

**Figure 7:**
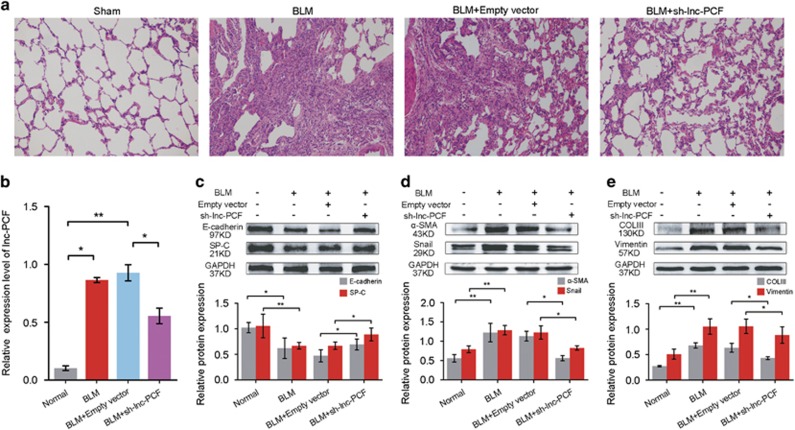
Lnc-PCF as a potential therapeutic target for pulmonary fibrosis. (**a**) sh-lnc-PCF could improve the alveolar structure *in vivo* through H&E staining, × 400 magnification. (**b**) Rats sprayed with sh-lnc-PCF showed decreased lnc-PCF expression compared with those in the BLM group based on qRT-PCR. (**c**) sh-lnc-PCF could promote E-cadherin and SP-C expression. (**d**) sh-lnc-PCF could inhibit *α*-SMA and Snail expression. (**e**) sh-lnc-PCF could inhibit collagen III and vimentin expression. Each bar represents the mean±SD, *n*=6, *P<0.05, **P<0.01

An interfered sequence of lnc-PCF(sh-lnc-PCF) was synthesized, packaged in adenovirus, and sprayed across rat lung tissues by using a Penn-Century MicroSprayer (Penn-Century Inc., Wyndmoor, PA, USA) to determine the potential of lnc-PCF as a therapeutic target *in vivo*. Fibrosis was evaluated using hematoxylin and eosin staining (H&E). The rats in the sh-lnc-PCF group presented a continuous bronchial mucous membrane structure with a more intact wall than those in the BLM group. The alveoli in the sh-lnc-PCF group showed clearer hollow cavities with thinner alveolar walls than those in the BLM group. In addition, the lung mesenchyme in the sh-lnc-PCF group displayed few collagen fibers, thereby indicating that the hallmark of the fibroblastic foci was distinctly decreased (Figure 7a). The BLM group had significantly increased lnc-PCF level compared with that in the sham group. Furthermore, sh-lnc-PCF could inhibit lnc-PCF expression in the sh-lnc-PCF group compared with that in the BLM group (Figure 7b).

To further investigate the antifibrotic action of sh-lnc-PCF *in vivo*, we tested the expression levels of E-cadherin, SP-C, *α*-SMA, collagen III, and vimentin, which are indicators of pulmonary fibrosis, after sh-lnc-PCF spraying *in vivo*. The sh-lnc-PCF could decrease the expression levels of a-SMA, collagen III, and vimentin, but increase those of E-cadherin and SP-C in the sh-lnc-PCF group as compared with those in the BLM group (Figures 7c–e).

## Discussion

**Figure 8:**
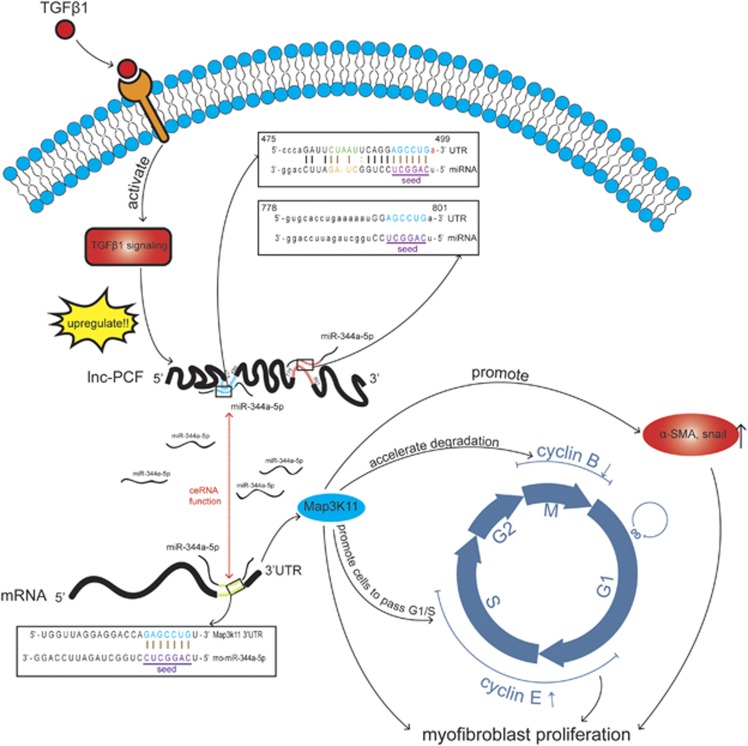
Lnc-PCF promoted the proliferation of epithelial cell activated with TGF-*β*1 by competitively binding with miR-344a-5p-targeted map3k11 in pulmonary fibrosis

In contrast to the role of miRNA, the function of lncRNA is significantly unknown. Emerging evidence suggests that the changes in lncRNAs are associated with the development of various diseases.^25, 26^ Several studies reported that lncRNA can act as ceRNA for miRNA and might be involved in physiological and pathological processes.^27, 28^ Kallen *et al.*^29^ reported that the increased expression of paternally imprinted H19 lncRNA may act as a sponge for let-7, thereby explaining the downregulation of this miRNA in non-small-cell lung cancer types. However, whether lncRNAs interact with miRNAs to form an interrelated regulatory network in IPF remains unknown.^30^ In the current study, we presented a novel, highly expressed lnc-PCF that can promote the proliferation of activated epithelial cells by competitively binding to miR-344a-5p-targeted map3k11 in pulmonary fibrosis (Figure 8). Although targeting the liver-specific miR-122 by using an antisense-based approach is not related to IPF, this method is currently under human trials for the treatment of the hepatitis C virus.^31^ Our findings suggested that lnc-PCF is a significant cause of IPF and can be used as a potential therapeutic target through different approaches, such as antisense oligonucleotides and short interfering RNAs.

The present study is a follow-up to a prior work in our laboratory, which revealed the differential expression of lncRNAs, analyzed the relationship between lncRNA and protein-coding gene, and identified the role of lncRNA as ceRNA in pulmonary fibrosis.^10, 11^ In the current study, lnc-PCF was identified as a highly expressed lncRNA associated with IPF development. Our result is consistent with several reports, which posited that the abnormally expressed lncRNAs are closely related to several important fibrotic diseases and can influence the expression and intracellular distribution of specific proteins.^32, 33, 34^ lncRNAs exhibit a distinct expression pattern both in time and space and are highly expressed in specific cells where they are activated.^35^ The number of foreseen lncRNA transcripts may even exceed the number of protein-coding mRNAs in mammals; thus, a growing body of evidence involved in lncRNA-based studies focuses on the pathogenesis of pulmonary fibrosis.^36, 37^ Although the ability to predict lncRNA was facilitated with genomic sequencing and bioinformatics analyses, this assumptive gene must be verified. In the present study, we combined microarray analysis and gene data bank and identified the sequence, ORF, locations on the chromosome, and translation activities of this gene *in vitro*. We concluded that BC158825 is a sense lncRNA, which is referred to as lnc-PCF. Our results showed that lnc-PCF is highly expressed *in vivo* and *in vitro*, which is consistent with our microarray analysis.

IPF is a severe disease that is characterized with myofibroblast proliferation. The transition of epithelial cells into myofibroblast is one of the main sources of myofibroblasts. In this process, epithelial cells lose their epithelial phenotype, acquire myofibroblast-like properties, and exhibit decreased cell adhesion and increased motility. This process is accompanied by an increase in pulmonary fibrosis markers, such as *α*-SMA, collagen, vimentin, and Snail, and has also gained considerable research attention in the past few years as a potential contributor to pulmonary fibrosis.^38, 39, 40^ Our data showed that lnc-PCF could stimulate the proliferation of myofibroblast derived from epithelial transition by facilitating the driving cell cycle progression. Nevertheless, the mechanism of lnc-PCF to regulate the transformation of epithelial cells into myofibroblast remains unknown. Different types of RNAs communicate with one another via their targeted miRNAs. This regulatory mode is involved in oncogenesis and cancer progression.^41, 42, 43, 44^ However, investigations on this regulatory mode were performed almost exclusively in non-small-cell lung cancer in respiratory diseases.^30^ Therefore, we further investigated the ability of lnc-PCF to regulate mRNA through miRNA in pulmonary fibrosis. In this study, lncRNA-miRNA, miRNA–mRNA, and mRNA–lncRNA interactions were thoroughly surveyed, identified, and detected. Systematic and integrative analyses of different RNA molecules with potential cross-talk may significantly contribute in determining the complex mechanisms underlying pulmonary fibrosis. Test screening and data analysis results showed that miR-344a-5p is one of the directly targeted miRNAs by lnc-PCF, and map3k11 is a target gene of miR-344a-5p. Lnc-PCF can regulate map3k11 by targeting miR-344a-5p to promote the proliferation of activated epithelial cells, which results in pulmonary fibrosis.

According to a clinical perspective, ncRNA targeted as a novel therapeutic approach will require a thorough understanding of their function and mechanism of action. In addition, the changes in the expression levels of miRNA and lncRNA can be used as biomarkers for disease strategy and/or assessment of drug action.^45^ In general, our study on the regulation of the activated epithelial cell proliferation in pulmonary fibrosis by lnc-PCF and miR-344a-5p-targeted map3k11 introduces a new approach to examine the complex post-transcriptional regulatory networks and present new therapeutic approaches to pulmonary fibrosis.

## Materials and methods

### Animal model and treatment

Sprague–Dawley rats with a mean weight of 200 g were obtained from the Green Leaf Experimental Animal Center (Yantai, China) and maintained according to the regulation approved by the Institutional Animal Ethics Committee of Binzhou Medical University.^46^ Pulmonary fibrosis was induced with a single intratracheal instillation of BLM dissolved in saline as previously described.^47^ The normal group was administered with an equivalent volume of saline. Lung tissues were collected on 7, 14, 21, and 28 days following BLM treatment.

The interfered sequence of lnc-PCF was packaged in the adenovirus. The treatment of adenovirus packaging interfered with the sequence of lnc-PCF was as follows: the rats were randomly divided into the following four groups (10 rats each): sham, BLM-treated (BLM), BLM+empty vector, and BLM+sh-lnc-PCF. The adenovirus packaging interfered with lnc-PCF sequence was sprayed into the rat lung tissues by using a Penn-Century MicroSprayer (Penn-Century Inc.). All rats were killed on day 28. Lung tissue sections were collected and immediately frozen in liquid nitrogen for further studies.

### Cell culture

RLE-6TN cell line was purchased from the Type Culture Collection of the Chinese Academy of Sciences (Shanghai, China) and maintained in DMEM medium (Sigma, St. Louis, MO, USA) supplemented with 10% fetal bovine serum and 2% penicillin and streptomycin under the condition of 37 °C and 5% CO_2_ atmosphere. This cell line was further used for experiments upon reaching 70–80%.

### RNA transfection

Lnc-PCF smart silencer, miR-344-5p mimic, and miR-344-5p inhibitor were purchased from RiboBio Co., Ltd. (Guangzhou, China). The RLE-6TN cells were plated in six-well culture dishes (2 × 10^5^ cells per well) until the cell density reached 70–80% prior to small RNA transfection. On the following day, the cells were transfected with 50 nM small RNA accompanied with reagent buffer by using a ribo FECT™CP Transfection Kit (Guangzhou RiboBio, Guangzhou, China) according to manufacturer’s instructions. After 6 h of culture at 37 °C, the complexes were replaced with complete medium. All cells were collected for Western blot or other experiments.

### qRT-PCR

Total RNA from collected cells was extracted using Trizol reagent. The growth media were briefly removed from the culture dish. Approximately 1 ml of TRIzol was directly added to each 9.5 cm^2^ well. Cell lysis was achieved by repeatedly pipetting the cells up and down. The cells were incubated at room temperature for 5 min. Afterward, 200 *μ*l of chloroform was added to each sample. The samples were shaken and incubated for 3 min at room temperature and subsequently centrifuged at 12 000 × *g* at 4 °C for 15 min. The samples were divided into three layers. The aqueous phase was reserved with 500 *μ*l of isopropyl alcohol at room temperature for 10 min. The samples were centrifuged at 10 000 × *g* at 4 °C for 10 min, and the supernatant was removed. The RNA pellet was gently washed with 500 *μ*L of ethanol, mixed, centrifuged at 7500 × *g* for 5 min at 4 °C, and resuspended in 10 *μ*l of RNA-free water. A Nanodrop 2000 ultraviolet spectrophotometer was used to determine the concentration and purity of the extracted RNAs. The single-stranded cDNAs of miRNA and lncRNA were synthesized from 2 *μ*g of RNA. Real-time fluorescence quantification PCR was performed with a SYBR green-based PCR Master Mix Kit (Takara, Shiga, Japan) on a Rotor Gene 3000 RT-PCR system of Corbett Research (Sydney, NSW, Australia). The PCR reaction system for miRNA was as follows: initial denaturation at 95 °C for 20 s and30 cycles of 95 °C for 10 s, 60 °C for 20 s, and 72 °C for 10 s. For lncRNA, the reaction system was as follows: initial denaturation at 95 °C for 30 s and 35 cycles of 95 °C for 5 s and 60 °C for 20 s. The threshold cycle was determined after the reactions. The relative miRNA and lncRNA expression levels were calculated based on *Ct* values and were normalized to the U6 or GAPDH levels of each sample, respectively.

### Immunofluorescence staining

Approximately 2 × 10^5^ RLE-6TN cells were cultivated on the sterile slides in a 24-well plate. After small RNA transfection and TGF-*β*1 treatment, these cells were rinsed with cold phosphate-buffered saline (PBS) for three times and fixed in 4% paraformaldehyde for 1 h. The cell lines were rinsed with PBS for three times, incubated with 0.5% Triton X-100 for 15 min at room temperature, and blocked with 10% normal goat serum for 1 h at 37 °C. Afterward, *α*-SMA antibody (1 : 300) was added dropwise into each slide at 4 °C overnight. RLE-6TN was rinsed with PBS for three times and incubated with fluorescein-labeled immunoglobulin G (IgG) antibody (1 : 200) *α*-SMA (FITC-labeled) at 37 °C for 1 h in a wet box. All operating steps were conducted in the dark because fluorescent secondary antibodies were added. After rinsing with PBS for three times, the nuclei were stained with DAPI (Roche Molecular Biochemicals, Basel, Swizerland) for 5 min at room temperature. Immunofluorescence was analyzed under a fluorescence microscope.

### Western blot

Cells were lysed with PIPA lysis buffer. A total of 30 *μ*g of protein was run on a 10% SDS-polyacrylamide gel. After being electrophoretically transferred to a pure nitrocellulose blotting membrane (Pall Life Sciences, Ann Arbor, MI, USA), the bolts were probed with primary antibodies, with GAPDH as an internal control. Exposure was performed using a chemiluminescence imaging system (Clinx Science Instruments, Shanghai, China). The bands were quantified by measuring the band intensity for each group.

### Flow cytometry analysis

Approximately 1 × 10^5^ cells were collected and resuspended in 500 *μ*l of 70% cold ethanol, which was added dropwise under gentle vortexing. The cells were fixed overnight at 4 °C, collected by centrifugation, washed once in PBS, collected again, and stained with propidium iodide (PI) staining solution, which was composed of 20 *μ*g/ml PI, 200 *μ*g/ml RNAse A, and 0.1% Triton X-100 for 15 min at 37 °C. The DNA contents of the stained cells were analyzed using a flow cytometer.

### Dual-luciferase reporter assay

Vector construction: we used the forward-sequenced primer (5′-GAGGAGTTGTGTTTGTGGAC-3′) and reverse-sequenced primer (5′-TGTAAAACGACGGCCAGT-3′) to obtain the lnc-PCF sequence. Restriction enzymes, namely, *Mlu*I and *Hind*III, were used to sever the lnc-PCF sequence at AAGCTT and ACGCGT, respectively. The digested lnc-PCF was inserted into the rear of the luciferase reporter genes (firefly and *Renilla*) in Pmir-REPORT-lncPCF pronucleus Amp resistance. Plasmid-transfected 293 T cells: 293 T cells were cultivated in a 96-well plate at a density of 2 × 10^5^/well. After 24 h, lnc-PCF and transfection reagents were prepared using 0.2 *μ*g of firefly, 0.01 *μ*g of *Renilla*, and 0.25 *μ*l of transfection reagents and were incubated for 5 min. MiR-344a-5p transfection reagents were prepared at the final density of 100 nM miRNA and 0.25 *μ*l of transfection regents and incubated for 5 min. Afterward, we mixed lnc-PCF and miR-344a-5p with their transfection reagents and incubated the mixtures for 20 min. About 50 *μ*l of the medium was removed, and 25 *μ*l of lnc-PCF transfection mixture and 25 *μ*l of miR-344a-5p transfection mixture were added to the cultured cells. After transfection for 6 h, the transfection mixture was replaced with a new complete medium. Dual-luciferase reporter assay: after cotransfection for 48 h, the complete medium was removed, and the cells were rinsed using 100 *μ*l of PBS, which was subsequently replaced with 1 × passive lysis buffer to lyse the cells. The mixture was shaken for 15 min. Each well was added with 100 *μ*l of Stop&Gloreagent for 2 s to obtain test data under dark condition.

### RNA pull-down

Cloning of the lnc-PCF sequence into Pgen-3z vector: the lnc-PCF sequence was ligated into multiple cloning sites located between the T7 promoter (5′-TAATACGACTCACTATAGGG-3′) and SP6 promoter (5′-AATTTAGGTGACACTATAGAA-3′). Approximately 5 *μ*l of restriction endonuclease was added to 20 *μ*g of plasmid. The total volume was adjusted to 50 *μ*l by using H2O. The mixture was incubated at 37 °C from 2 h to overnight. The linearized plasmid was purified using the QIAquick Gel Purification Kit. After purification and size check of RNA, the prepared denatured RNA gel was used to confirm whether or not the RNAs were transcribed at the right size. Sufficient cells were cultured and collected at a minimum of 10 cm × 10 cm plate for each pull down. The cell pellet was suspended using 1 mL of ProteaPrepZwitterionic cell lysis buffer supplemented with protease and phosphatase inhibitor cocktails (1 : 100), anti-RNase (1U/*μ*l), panobinostat (1 : 100), and methylstat (1 : 100). The tube was then incubated on ice for 40 min and vortexed every 10 min. The mixture was centrifuged at 14 000 × *g* for 15 min at 4 °C, and the supernatant was transferred to a new tube for further use. Afterward, 50 *μ*l of beads were transferred to a fresh tube. The tube was placed on a magnetic separator for 1 min, and the supernatant was removed. After clearing the lysate with activated beads to reduce nonspecific binding, 20 *μ*g of biotinylated RNA was heated to 90 °C for 2 min. The mixture was chilled on ice for 2 min and briefly centrifuged. The total volume was adjusted to 100 *μ*l by using the RNA structure buffer. The mixture was incubated at room temperature for 20 min to allow proper secondary structure formation. About 50 *μ*l of activated avidin magnetic beads were also prepared. The beads were immediately subjected to RNA (20 *μ*g) capture in RNA capture buffer for 30 min at room temperature with gentle agitation. The RNA capture buffer was removed. The RNA-captured beads were washed once with NT2 buffer and incubated with 30 mg pre-cleared cell lysate for 2 h at 4 °C with gentle rotation. The tube was centrifuged and placed on a magnetic separator. The supernatant was removed from the beads and discarded. The RNA-binding protein complexes were washed with NT2 buffer twice, NT2 high-salt buffer (500 mM NaCl) twice, NT2 high-saltbuffer (1 M NaCl) once, NT2 high-saltbuffer (750 mM KSCN) once, and PBS twice. The beads were eluted by incubating the sample with elution buffer for 20 min at 4 °C with frequent agitation, and the elute was collected. The elution step was repeated, and the elutes were pooled together. PCR was used to test the samples.

### RIP experiment

RIP experiment was performed using an EZ Magna RIP kit (Millipore, Billerica, MA, USA) following the manufacturer’s protocol. The cells were lysed in complete RIP lysis buffer. The extract was incubated with Ago2 antibody or control IgG (Millipore, Billerica, USA, MA) 6 h at 4 °C, followed by adding magnetic beads conjugated with proteinA/G (Thermo Fisher Scientific, MA, USA). The beads were washed and incubated with Proteinase K to remove the proteins. Finally, purified RNA was subjected to qRT-PCR analysis using specific primers for lnc-PCF.

### *In vitro* translation assay

The T7-BC158825 sample DNA was prepared by PCR using the following primers: T7-T7-BC158825-F:5′-ACCGCCTAATACGACTCACTATAGGGACCTCCACCTGCATGACCCTGGC-3′T7-BC158825-R:5′-TTTTTTTTTTTTTTTTTTTTTTTTTTTTTTACTGAGTGAATAAATGAGCCAATAT-3′. The PCR product was purified using a Gel Extraction Kit (Transgen Biotech, Beijing, China). The reagents were removed from storage at −70 °C. The TnT Quick Master Mix (Promega, Madison, WI, USA) was rapidly thawed by hand-warming and placing on ice. Other components were thawed at room temperature and stored on ice. Following the component chart above, we assembled the reaction components in a 0.5 or 1.5 ml microcentrifuge tube. When all of the components were added, the mixture was gently mixed by pipetting. If necessary, the mixture was centrifuged briefly to return the reaction to the bottom of the tube. We included a control reaction with no added DNA, that is, H_2_O in this work. This reaction allowed the measurement of any background incorporation of labeled amino acids. T7 Luciferase Control DNA was used as positive control. The reaction was incubated at 30 °C for 60–90 min, and the translation results were analyzed. After SDS-PAGE electrophoresis, the gel was stained using InstantBlue Ultrafast Protein Staining Solution (Expedeon Ltd. 25 Norman Way, Over Cambridgeshire CB24 5QE, UK).

### Statistical evaluation

Data were statistically analyzed in SPSS version 19.0 (IBM, Armonk, NY, USA). Data were presented as mean±S.D. of at least three independent experiments. Unpaired Student’s *t*-test was used to compare the two groups. One-way ANOVA with Student–Newman–Keuls *post hoc* test was conducted to compare three or more groups. Statistical significance was considered at *P*<0.05.

## Publisher’s Note

Springer Nature remains neutral with regard to jurisdictional claims in published maps and institutional affiliations.
