# Supplementary figures and images for: A novel lnc-PCF promotes the proliferation of TGF-β1-activated epithelial cells by targeting miR-344a-5p to regulate map3k11 in pulmonary fibrosis
Source: Cell Death Dis. 2017 Oct 26;8(10):e3137–. doi: 10.1038/cddis.2017.500 (PMC5682666; doi:10.1038/cddis.2017.500)

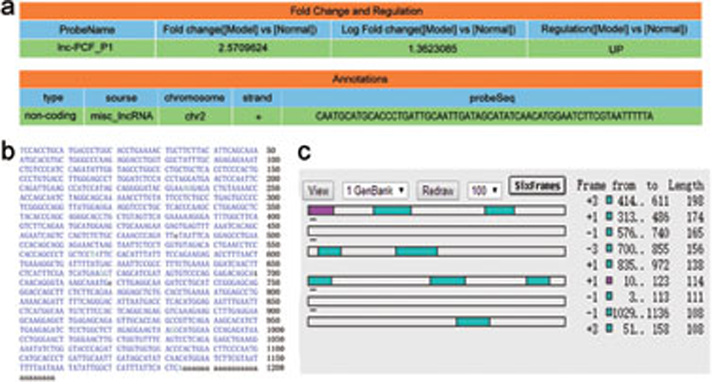

Supplement: Supplementary Figure 1 [file cddis2017500x1.tif]

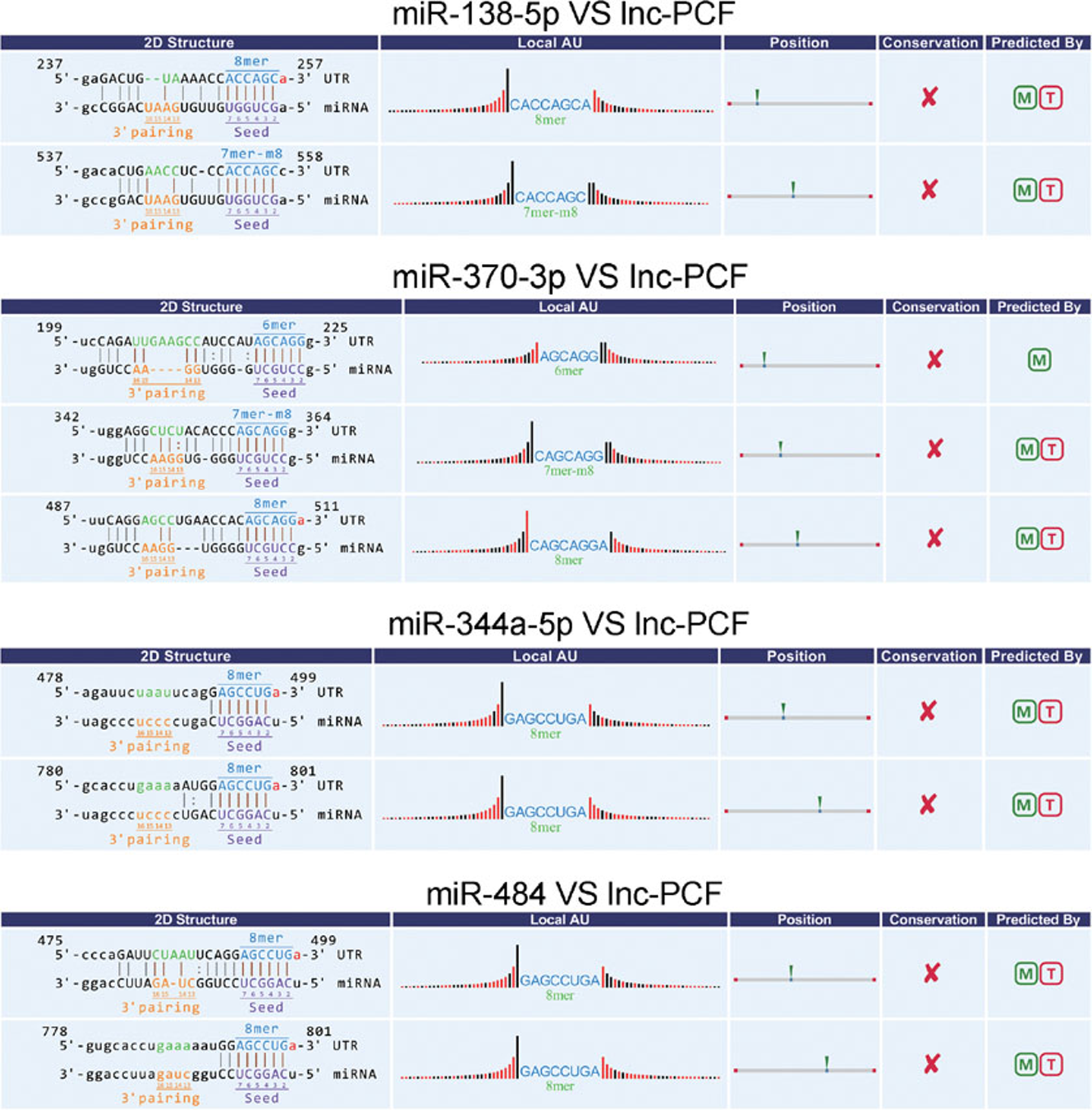

Supplement: Supplementary Figure 2 [file cddis2017500x2.tif]
