# Supplementary material for: A novel lnc-PCF promotes the proliferation of TGF-β1-activated epithelial cells by targeting miR-344a-5p to regulate map3k11 in pulmonary fibrosis
Source: Cell Death Dis. 2017 Oct 26;8(10):e3137–. doi: 10.1038/cddis.2017.500 (PMC5682666; doi:10.1038/cddis.2017.500)
Supplement: Supplementary Figure Legends [file cddis2017500x3.docx]

**SUPPLEMENTARY FIGURE LEGENDS**

**Supplementary figure 1** Bioinformatics analysis of lnc-PCF. (a) BC158825 was expressed more than three times the level in pulmonary fibrosis and classified as lncRNA by using ArrayStar lncRNA microarray analysis. We renamed BC158825 as lnc-PCF based on its function, that is, lnc-PCF can promote the proliferation of activated epithelial cell during pulmonary fibrogenesis. (b) Full-length lnc-PCF, including 1208 bp, was acquired from the UCSC database. (c) Open-reading frame analysis further verified the no-coding protein ability of lnc-PCF.

**Supplementary figure 2** 2D structures of miR-138-5p, miR-370-3p, miR-484, and miR-344a-5p binding sites on the lnc-PCF transcript. Four miRNAs were selected to analyze their affinities with lnc-PCF, including their seed sequences (marked by color), local AU (a red bar indicates an improved AU), binding position (with a close location to the two sides being preferable), and conservation.
